# Mass social contact interventions and their effect on mental health related stigma and intended discrimination

**DOI:** 10.1186/1471-2458-12-489

**Published:** 2012-06-28

**Authors:** Sara Evans-Lacko, Jillian London, Sarah Japhet, Nicolas Rüsch, Clare Flach, Elizabeth Corker, Claire Henderson, Graham Thornicroft

**Affiliations:** 1 Health Service and Population Research Department, King’s College London, Institute of Psychiatry, 29, De Crespigny Park, London, SE5 8AF, UK; 2 Applied Social Science, University of Sussex, School of Psychology, Brighton, UK; 3 Department of Social and General Psychiatry, Psychiatric University Hospital Zürich, Zürich, Switzerland

**Keywords:** Stigmatization, Mental Disorders, Behaviour, Social contact, Health Promotion

## Abstract

**Background:**

Stigma and discrimination associated with mental health problems is an important public health issue, and interventions aimed at reducing exposure to stigma and discrimination can improve the lives of people with mental health problems. Social contact has long been considered to be one of the most effective strategies for improving inter-group relations. For this study, we assess the impact of a population level social contact intervention among people with and without mental health problems.

**Methods:**

This study investigated the impact of social contact and whether presence of specific facilitating factors (equal status, common goals, cooperation and friendship potential): (1) improves intended stigmatising behaviour; (2) increases future willingness to disclose a mental health problem; and (3) promotes behaviours associated with anti-stigma campaign engagement. Two mass participation social contact programmes within England’s *Time to Change* campaign were evaluated via a 2-part questionnaire. 403 participants completed initial questionnaires (70% paper, 30% online) and 83 completed follow-up questionnaires online 4–6 weeks later.

**Results:**

This study investigated the impact of social contact and whether presence of specific facilitating factors (equal status, common goals, cooperation and friendship potential): (1) improves intended stigmatising behaviour; (2) increases future willingness to disclose a mental health problem; and (3) promotes behaviours associated with anti-stigma campaign engagement. Two mass participation social contact programmes within England’s *Time to Change* campaign were evaluated via a 2-part questionnaire. 403 participants completed initial questionnaires (70% paper, 30% online) and 83 completed follow-up questionnaires online 4–6 weeks later. Campaign events facilitated meaningful intergroup social contact between individuals with and without mental health problems. Presence of facilitating conditions predicted improved stigma-related behavioural intentions and subsequent campaign engagement 4–6 weeks following social contact. Contact, however, was not predictive of future willingness to disclose mental health problems.

**Conclusions:**

Findings emphasise the importance of facilitating conditions to promote positive social contact between individuals and also suggest that social contact interventions can work on a mass level. Future research should investigate this type of large scale intervention among broader and more representative populations.

## Background

Social contact has long been considered to be one of the most effective strategies for improving inter-group relations [1,2]. Several studies support the social contact hypothesis in reducing stigma against people with mental health problems [3-6]. Despite the effectiveness of this approach, barriers to social contact between people with and without mental health problems persist. The more people believe that they are or will be stigmatized, the more they will conceal their mental illness and withdraw from contact [7,8], thus reducing opportunities for intergroup contact. Thus, to achieve positive social contact interactions, we also need to reduce barriers to and provide opportunities for ‘normalising’ disclosure.

Disclosure is an important element of the social contact interaction [9] as one’s mental health status is not a visibly apparent characteristic. Additionally, self-disclosure may be a key factor in facilitating positive social contact via increasing intimacy and potential for friendship [10]. A positive environment which supports the disclosure of mental health problems in addition to training and strategies for managing disclosure of one’s own mental health problems may be important for reducing barriers to positive social contact. An atmosphere of normalized disclosure in which the discloser and those disclosed to can interact in a comfortable social situation could improve conditions for both the ingroup and outgroup by facilitating reflection among both groups and reducing paradigms of thinking using ‘us vs. them’ [11,12]. We know that at the macro level, people with mental illness living in countries where the general public feel more comfortable talking to people with mental health problems reported lower levels of self-stigma and perceived discrimination and higher levels of empowerment. Thus, intergroup contact may facilitate a virtuous cycle leading to subsequent reduction in public stigma and more favourable self-appraisals by individuals with mental health problems.

In addition to disclosure, other considerations should be made when considering how to optimise social contact. There is much discussion around the conditions which can maximise the effect of social contact. In 1954, Gordon Allport suggested 4 factors for reducing prejudice: (1) Equal status between the groups in the situation; (2) Common goals; (3) Intergroup cooperation and (4) Support of authorities, law, or custom [1]. Allport’s hypothesis was later extended by Pettigrew [13] with the additional factors of time and “friendship potential.” A meta-analysis performed by Pettigrew and Tropp, including 66 samples from studies focused on mental illness, supports the conception that contact is effective in reducing prejudice among a broad range of groups [14]. Moreover, the meta-analysis showed that studies which structured the contact intervention to meet Allport’s conditions had a higher mean effect size compared to samples which did not. Despite the positive effects associated with these factors, evidence from the meta-analysis and other studies suggests that the elements put forth are not necessary conditions but rather ‘facilitating conditions’ of positive contact [14].

*Time to Change (TTC)*[15,16] is England’s largest ever anti-stigma campaign which aims to reduce stigma and discrimination against people with mental health problems. As one of its approaches to reducing stigma and discrimination, TTC applies the theoretical concepts of social contact at the population level via mass level social contact events [17]. These social contact events aim to engage members of the public with and without mental health problems and to reduce stigma and discrimination against people with mental health problems. This study examines the effectiveness of these events in facilitating positive social contact and their subsequent impact on stigma and discrimination. Specifically, we examine whether experience of social contact at TTC events: (1) improves intended stigmatising behaviour; (2) increases future willingness to disclose a mental health problem; and (3) promotes behaviours associated with campaign engagement.

## Methods

### Intervention

TTC incorporated two types of mass social contact interventions: The *Roadshow* and *Time to Get Moving*. These interventions aim to attract members of the public with and without mental health problems and to facilitate positive intergroup contact and disclosure of mental health problems, in an effort to break down the stigma surrounding mental health problems.

The TTC *Roadshow* is aimed at engaging the public at a grassroots level. The Roadshow included a series of 12 events which ran from 21^st^ September to 17^th^ October 2009. For each Roadshow event, a stand was set up in a prominent town centre location with high footfall (e.g., high streets, shopping centres and political party conferences). The stand was run by TTC staff and staff from local organisations including 100 volunteers with direct experience of mental health problems. Volunteers aimed to engage the public with TTC campaign materials, e.g., postcards, scratch cards etc, and all participants were asked to join the ‘visual pledge’ by having a photo taken or producing a written message or video.

*Time to Get Moving* consists of over 200 mass participation physical activity events that take place throughout England during one week in October of each year (around World Mental Health Day). These events are advertized throughout the community as a call to action against mental health stigma. Events include, e.g., football matches, leisure centre days, dance workshops, and walking groups and provide an opportunity for sociable activity. Events aim to bring together diverse members of the community both with and without experience of mental health problems to participate in various activities in an informal, real world setting.

TTC volunteers with direct experience of mental health problems were present at Time to Get Moving and Roadshow events to educate participants about mental health issues and engage the public in activities. Prior to the event, volunteers undertook training to build their confidence in disclosing and discussing their experience of mental health problems. At some events, celebrities also spoke and pledged their support to combat mental health-related discrimination or spoke about their own experiences of mental distress.

### Participants

Participants were recruited from a selection of 4 geographically representative Time to Get Moving events and all 12 Roadshow events. All participants over the age of 18 attending an event were eligible to participate. A questionnaire (more detail on questionnaire and measures included below) was given to participants at the end of the event or after they finished participating in the activity. Participants were allowed to fill out the questionnaire on their own in private and placed their completed questionnaires in a covered box before leaving the event site. Participants were also given the opportunity to fill out the questionnaire online via a website address included in the event information packs. Participants who filled out the initial questionnaire were asked to provide their contact details to participate in a follow-up questionnaire. Individuals who participated in the follow-up were entered into a prize draw.

A total of 403 individuals filled out an initial questionnaire (70% paper, 30% online). All follow-up questionnaires were filled out online 4–6 weeks following completion of the initial questionnaire. 50% of participants provided their contact details to fill out a follow-up questionnaire and 40% of those individuals (n = 83) filled out a follow-up questionnaire online.

### Questionnaire

The questionnaire assessed presence and quality of social contact and its relationship with three stigma related outcomes. Assessment of social contact and stigma related outcomes are discussed in detail below. Participants were also asked to provide demographic information and basic contact details for follow-up.

### Presence and quality of social contact

Presence of social contact was assessed by identifying whether the participant met someone from the *opposite* group (i.e., did people with mental health problems meet someone without mental health problems, ‘discloser’ at the event and vice versa ‘those being disclosed to’). Quality of social contact was assessed among individuals who met someone from the opposite group by inquiring about the presence of the specific facilitating conditions theorised by Allport and Pettigrew [1,13]. Specifically, individuals were asked to ‘describe their meeting’ to investigate the following factors: (1) Equal status between the groups in the situation (I felt like we were equals in the conversation); (2) Common goals (Do you feel like you were both able and willing to achieve that goal?); and (3) Intergroup cooperation (During the event did you feel like you were actively working together (i.e., by getting involved in an activity, or to reduce discrimination based on mental illness, or to gain a better understanding of mental health problems?) and (4) Friendship Potential (Do you feel you got to know the person?). For these events, support of authorities, law, or custom to further understand what constitutes meaningful social contact was assumed. Following item development, review and judgement of items were performed by members of the TTC Lived Experience Advisory Panel (mental health service users with research experience), members of the mental health charities Mind and Rethink conducting the intervention, and international experts in the field of social contact and stigma research. The panel was asked to ensure that the framework addressed the elements hypothesised by Allport and Pettigrew and assessed face validity. Cognitive testing/interviewing was then performed on a sample of 10 laypersons at a TTC event in Taunton, England. After filling out the survey, individuals were interviewed to elicit feedback specifically about wording, comprehensibility, and response format.

### Stigma related outcomes

#### Intended behaviour

Intended behaviour (the level of intended future contact with people with mental health problems) was measured by the RIBS [18]. We assessed changes in 4 outcomes: living with, working with, living nearby and continuing a relationship with someone with a mental health problem. Overall test-retest reliability of the RIBS is 0.75 and overall internal consistency is 0.85.

#### Willingness to disclose a mental health problem in the future

Whether participants felt comfortable to disclose a mental illness to friends or relatives was measured by another item (‘In general, how comfortable would you feel talking to a friend or family member about your mental health, for example telling them you have a mental health diagnosis and how it affects you?'). Seven response options range from strongly agree to strongly disagree.

#### Engagement with TTC campaign

At follow-up, participants were asked whether they had engaged in TTC related activities such as: visiting the website, pledging support via the TTC visual pledge, talking with others about the event, or following TTC on Facebook or Twitter.

### Analysis

Survey responses were described and categorised based on experience of mental health problems. Characteristics of initial and follow-up respondents are presented and comparisons were made between initial respondents who did and did not complete a follow-up using a chi-square test. Change scores were calculated for RIBS items and disclosure items. Respondents were categorised based on whether their score improved at follow-up compared to initial survey (yes/no). Separate logistic regression models examined the effect of social contact on change in RIBS score, change in willingness to disclose and subsequent campaign engagement. Initial models tested individual elements of social contact. Following this, a summary model was fitted which included an aggregate measure summing all social contact factors (1 point for presence of each factor). Models controlled for sociodemographic characteristics (gender, age, race/ethnicity), previously knowing someone with a mental health problem, initial RIBS score and initial willingness to disclose score. Type of intervention, i.e., Roadshow or Time to Get Moving, were included as a regression covariate to see if type of event was related to outcome; however, as this variable was not significant, it was not included in the final model.

Analyses were carried out using Stata version 10 and SAS version 9.1. This study was approved by the King’s College London, Psychiatry, Nursing and Midwifery Research Ethics Subcommittee.

## Results

These results consist of data collected from the 403 participants (estimated 6–10% of all event participants) who completed an initial questionnaire and 83 participants who completed a follow-up questionnaire. About two-thirds (61%, initial and 64%, follow-up) reported having a mental health problem.

The average age of participants who completed the initial questionnaire was 38 years old. The majority of participants were white (83%) and female (64%). There were no significant differences in these characteristics among initial or follow-up respondents or among people with or without mental health problems (See Table 1). 16% of people at initial survey and 22% of respondents at follow-up stated that they did not know anyone with a mental health problem; however, this difference was not statistically significant (Z score = −1.33 p = 0.18).

**Table 1:** Characteristics of people with and without mental health problems (MHP)

	People with MHP (n = 257) n (%)	People w/o MHP (n = 146) n (%)
Age		
Mean (s.d.)	38.1 (13.4)	36.9 (14.1)
Gender		
Female	165 (64.5)	93 (64.1)
Ethnicity		
Asian	19 (7.4)	9 (6.4)
Black	10 (3.9)	9 (6.4)
Mixed	12 (4.7)	5 (3.6)
White	213 (83.2)	118 (83.7)
Other	2 (0.8)	0 (0.0)
Social Contact		
Self	257 (100.0)	0 (0.0)
Partner	8 (5.1)	5 (5.2)
Family	4 (2.6)	0 (0.0)
Friend	21 (13.4)	13 (13.5)
Work Colleague	10 (6.4)	10 (10.4)
Acquaintance	55 (35.0)	26 (27.1)
Other	35 (22.3)	26 (27.1)
None	24 (15.3)	16 (16.7)
Total RIBS Score		
Mean (s.d.)	17.3 (4.4)	16.6 (4.4)
Disclosure to family/friend		
Very comfortable	105 (47.3)	53 (43.8)
Moderately comfortable	52 (23.4)	30 (24.8)
Fairly comfortable	23 (10.4)	21 (17.4)
Neither comfortable nor uncomfortable	5 (2.3)	6 (5.0)
Moderately uncomfortable	19 (8.6)	6 (5.0)
Very uncomfortable	18 (8.1)	5 (4.1)
Met someone from opposite group (with or without MHP)	102 (58.0)	65 (47.5)

### Social contact

Among participants with mental health problems, 49% disclosed that they had a mental health problem during the event. *Intergroup contact* was also present. 58% of participants with a mental health problem reported that they met someone *without* a mental health problem during the event. 48% of participants without a mental health problem reported that they met someone *with* a mental health problem at the event.

Table 2 describes the intergroup interaction among those who met someone with/without mental health problems at the event. The most common reason for knowing the person did/did not have a mental health problem was that the person told them. The most common facilitating condition present among people with and without mental health problems was intergroup cooperation, followed by common goals, equal status, and friendship potential. Individuals were asked about their memory of the interaction at follow-up. The majority of people (67%) without mental health problems remembered their interaction ‘very well’. This was significantly greater than the proportion of individuals with mental health problems (29%).

**Table 2:** Quantity and Quality of Social Contact (those who met someone with/without mental health problems [MHP])

	People with MHP ^a^ (n = 102) n (%)	People w/o MHP (n = 65) n (%)	Fisher’s Exact Test (p-value)
1. How did you know the person you met did/did not have a mental health problem			
They told me	38 (42.2)	26 (44.8)	0.42
Someone else told me	2 (2.2)	5 (8.6)	0.08
I made an assumption	29 (32.2)	13 (22.4)	0.15
Other	21 (23.3)	14 (24.1)	0.52
^b^2. Presence of facilitating conditions^c^ (agree strongly)			
Equal Status	35 (36.5)	15 (31.3)	0.08
Common Goals	43 (42.2)	26 (40.0)	0.46
Intergroup cooperation	45 (44.1)	28 (43.1)	0.52
Friendship potential	23 (22.6)	8 (12.3)	0.07
3. How long did you speak with the individual you met? (asked at follow-up)			
Less than 1 minute	6 (15.8)	0 (0.0)	0.17
1–5 minutes	18 (47.4)	5 (41.7)	0.32
5–10 minutes	5 (13.2)	3 (25.0)	0.50
10+ minutes	9 (23.7)	4 (33.3)	0.38
4. How well do you remember your conversation? (asked at follow-up)			
Very well	10 (26.3)	8 (66.7)	*0.01
A little bit	18 (47.4)	4 (33.3)	0.30
Do not remember	10 (26.3)	0 (0.0)	*0.05
5. Actions since event (asked at follow-up)			
Talked with others about event	5 (8.2)	2 (9.1)	0.68
Visited website	29 (47.5)	10 (45.5)	0.16
Joined Pledge	10 (16.4)	4 (18.2)	0.54
Facebook/twitter	11 (18.0)	2 (9.1)	0.30
None	11 (28.9)	2 (20.0)	0.33

^a^ 102 people with mental health problems and 65 without mental health problems answered questions 1 and 2. The sample size was lower for questions 3–5 (n = 61 for people with mental health problems and n = 22 for people without mental health problems)

^b^ can tick more than one

^a^ facilitating social contact conditions refer tothe specific conditions theorised by Allport and Pettigrew as being associated with optimal social contact.

* p <0.05

#### Predictors of improved RIBS score

Table 3 presents predictors of improved RIBS score following the event. Specifically, Table 3 provides the probability odds of improved RIBS score (i.e., ratio of the probability of improved RIBS score to the probability of an unimproved RIBS score)

**Table 3:** Predictors of Improved RIBS Score (binary yes/no) (n = 83)

	Individual social contact elements	Individual social contact elements	Individual social contact elements	Individual social contact elements	Additive social contact
	OR(95% CI)	OR(95% CI)	OR(95% CI)	OR(95% CI)	OR(95% CI)
Age	1.1 (1.0, 1.1)	1.1 (1.0, 1.1)	1.1(1.0, 1.1)	1.1 (1.0, 1.1)	1.1 (1.0, 1.1)
Gender	1.5 (0.2, 9.9)	2.0 (0.3, 2.8)	1.6 (0.2, 0.7)	1.8 (0.3, 2.7)	1.6 (0.2, 1.0)
BME	5.6 (1.0, 31.5)	6.1 (1.1, 33.3)	6.7 (1.1, 9.2)	6.0 (1.1, 3.1)	6.1 (1.1, 4.5)
Experience of Mental Health Problems (yes/no)	2.0 (0.4, 10.0)	2.7 (0.5, 13.6)	2.5 (0.5, 12.4)	2.1 (0.4, 10.8)	2.3 (0.5, 11.6)
Facilitating Social contact Factors^a^					
Equal Status Common	2.4 (0.7, 7.7)	--	--	--	--
Goals	--	2.0 (0.9, 4.7)	--	--	--
Intergroup cooperation	--	--	*2.5 (1.1, 5.9)	--	--
Friendship potential	--	--	--	2.8 (0.6, 11.8)	--
Quality of Social contact (Number of factors)	--	--	--	--	*1.4 (1.0, 1.8)
Initial RIBS Score	1.0 (0.8, 1.3)	1.0 (0.8, 1.3)	1.0 (0.8, 1.3)	1.0 (0.8, 1.3)	1.0 (0.8, 1.3)
Initial willingness to disclose	1.0 (0.6, 1.5)	1.0 (0.6, 1.5)	1.0 (0.6, 1.5)	1.0 (0.6, 1.5)	1.0 (0.6, 1.5)

p < 0.05

^a^facilitating social contact conditions refer tothe specific conditions theorised by Allport and Pettigrew as being associated with optimal social contact

Initial models present facilitating conditions (equal status, common goals, intergroup cooperation, and friendship potential) as individual elements while the final model investigates the additive effect of each contact element. One facilitating factor, intergroup cooperation, was a significant predictor of improved RIBS score at follow-up (Odds Ratio [OR]: 2.5, 95% Confidence Interval [CI]: 1.1, 5.9). Number of facilitating social contact factors present was also a significant predictor of improved RIBS score at follow-up (OR: 1.4, 95% CI: 1.0, 1.8). Initial RIBS score, sociodemographic factors, or experience of mental health problem were not associated with an improved RIBS score.

#### Predictors of subsequent campaign engagement

Table 4 presents predictors of engaging in subsequent behavioural actions related to the campaign (yes/no binary outcome). Specifically, Table 4 provides the probability odds of subsequent campaign engagement. Regarding individual social contact elements, equal status was an independent predictor of subsequent campaign engagement (OR: 7.0, 95% CI: 1.6, 31.3). Number of facilitating social contact factors was also associated with being more likely to engage in behaviour supporting the TTC campaign (OR: 1.4, 95% CI: 1.0, 1.9). Respondents with a higher initial RIBS score were also consistently more likely to engage in subsequent behaviour supporting the TTC campaign (additive model: OR: 1.4, 95% CI: 1.0, 1.7).

**Table 4:** **Predictors of subsequent behavioural actions engaging with campaign**^**a**^(binary yes/no) (n = 83**)

	Individual social contact elements	Individual social contact elements	Individual social contact elements	Individual social contact elements	Additive social contact
	OR(95% CI)	OR(95% CI)	OR(95% CI)	OR(95% CI)	OR(95% CI)
Age	1.0 (1.0, 1.1)	1.0 (1.0, 1.1)	1.0 (0.9, 1.1)	1.0 (0.9, 1.1)	1.0 (1.0, 1.1)
Gender	0.4 (0.1, 2.0)	0.7 (0.1, 3.6)	0.6 (0.1, 2.9)	0.6 (0.1, 3.0)	0.6 (0.1, 2.9)
BME	0.6 (0.1, 3.8)	0.7 (0.1, 3.7)	0.7 (0.1, 3.8)	0.8 (0.2, 3.8)	0.7 (0.1, 3.7)
Experience of Mental Health Problems (yes/no)	0.8 (0.2, 3.6)	1.2 (0.3, 5.4)	1.1 (0.2, 4.7 )	1.1 (0.3, 4.6)	1.0 (0.2, 4.6)
Specific Social contact Factors					
Equal Status	*7.0 (1.6, 31.3)	--	--	--	--
Common Goals	--	2.3 (0.9, 5.8)	--	--	--
Intergroup cooperation	--	--	1.9 (0.8, 4.5)	--	--Sus
Friendship potential	--	--	--	1.1 (0.3, 4.6)	--
Quality of Social contact (Number of factors)	--	--	--	--	*1.4 (1.0,1.9)
Initial RIBS Score	*1.3 (1.0, 1.7)	*1.4 (1.1, 1.8)	*1.4 (1.0, 1.8)	*1.4 (1.0, 1.8)	*1.4 (1.0, 1.7)
Initial willingness to disclose	0.8 (0.5, 1.2)	0.9 (0.6, 1.3)	0.8 (0.6, 1.3)	0.9 (0.6, 1.3)	0.8 (0.6, 1.3)

*p <0.05

^a^subsequent behavioural actions engaging with campaign refer to the following actions: visiting the TTC website, pledging support via the TTC visual pledge, talking with others about the TTC event, or following TTC on Facebook or Twitter

#### Predictors of disclosure

There were no significant factors, however which predicted an increase in reported propensity to disclose a mental health problem either at work or to family and friends (number of factors, OR: 1.2, 95% CI: 0.8, 1.8).

## Discussion

These data suggest that the TTC events may have facilitated inter-group contact between people with and without mental health problems in a meaningful way. Importantly, presence of social contact facilitating conditions were associated with improved behavioural intentions and subsequent campaign engagement 4–6 weeks following the interaction even when controlling for previously knowing someone with a mental health problem and initial RIBS score. Positive social contact, however, was not associated with future willingness to disclose mental health problems.

It is likely that the events both incorporate the factors hypothesised as facilitating conditions and provide an atmosphere conducive to disclosure. The fact that the events explicitly focused on mental health may have also increased the effectiveness of social contact. Other research has shown that increased salience increases positive contact with individuals and improves intergroup relations [19].

Interestingly, individuals without mental health problems remembered their interaction better than individuals with mental health problems suggesting that the event was more salient for those without mental health problems. Other research supports that hearing about personal experiences directly from people with mental health problems can have a significant effect on people without mental health problems at follow-up. Pinfold et al., showed that 42% of police officers who received a workshop which incorporated talks from carers and service users reported that they would remember the personal experiences that were delivered during the workshop (compared to 6% who recalled pieces of information) [20]. Data from this study, however, suggest that the findings apply to both groups, i.e. regardless of whether they have a mental illness or not. Given that the majority of the sample had experience of mental health problems, empowerment, encouragement to disclose, encouragement to participate in anti-stigma efforts and reducing social distance toward other ingroup members are important outcomes for this group in addition to reducing public stigma among participants without experience of mental health problems [21,22]. It is also important to note that these data did not suggest any worsening of outcomes as other studies, have shown mixed results including some adverse outcomes associated with certain types of contact [23].

### Limitations

A strength of this study is that we looked at a change in outcomes following a specific intervention associated with a specific interaction which was facilitated in a naturalistic setting. While this study provides new and important information supporting the use of large scale public health interventions to reduce stigma and discrimination through social contact, low response rates limit the generalizability of the findings. About 10% of individuals who participated in the events also completed a baseline form and about 20% of those participants also completed a follow-up questionnaire and this may have underpowered some of the analyses. Future research, might examine differences in greater detail among subgroups, especially between individuals with and without mental health problems, using a larger sample. Further, those with stronger prejudice may be less likely to attend the event while individuals who do attend the events may be more receptive to interacting with the ‘outgroup’. The surveys suggest that 15% of people without mental health problems reported not knowing anyone with a mental health problem and this is lower than among the general population (38% according to the most recent Attitudes to Mental Illness Survey in England). Nevertheless, although respondents may not be representative of the general public, the associations found in the regression equations remain valid and interesting and the findings of two significant and corroborative outcomes support the effectiveness of this large scale intervention. Replicating these results in a larger more representative sample and inclusion of a control group would help us better understand the effectiveness of this type of intervention among the general public or relative effectiveness among those with higher levels of prejudice and discriminatory behaviours. Additionally, investigation of these findings among diverse ethnic groups which may reflect diverse attitudes and allow for further investigation of multiple stigma (e.g., racism and mental health problems) could be important [24,25]. Further specificity of these findings and how they may vary by type of mental illness might also be important [26,27]. Our data are based on an initial survey and a follow-up survey. Assessment of stigma related outcomes before the event would be optimal; however, as the events were free and open to the public, we were unable to contact or recruit study participants before the events. Social desirability may have also influenced responses; however, we would expect social desirability for intended behaviours, to be higher at initial survey (as these forms were filled out in a public place) compared to follow-up. This would suggest that improvements in RIBS score would be underestimated. Finally, collinearity between the facilitating conditions make it difficult to estimate the independent effect of each facilitating condition; however, the lack of an association between most single elements still suggests that the number of facilitating conditions present is the most important factor.

## Conclusions

This study synthesises a combination of data from several interventions that take place in real world settings and that are part of a large anti-stigma campaign, and also incorporates information on variables such as social contact that are theoretically interesting. Additionally, we have developed a new evaluative approach which should further our understanding of what works in real life situations. .Findings emphasise the importance of facilitating conditions to promote positive contact and also suggest that social contact interventions can work on a mass level. Future work should build on this research by investigating large scale interventions among a broader and more representative population with larger sample sizes and incorporate use of control groups. Additionally, the role and characteristics of disclosure in relation to positive social contact might be further investigated. As there are different types and levels of disclosure which may sometimes result in negative reactions, we must be careful not to generalise all types of disclosure or these experiences outside of a supportive event such as the TTC events. It is likely that as the broader environment becomes more accepting of mental health problems, however, that the type and level of social contact will broaden [28]. As more individuals feel empowered to disclose their diagnosis, awareness of mental health problems should also increase, leading to a reduction in mental health stigma overall.

## Competing interests

The authors declare that they have no competing interests.

## Authors’ Contributions

SEL oversaw data collection, drafted the manuscript and conducted the analysis. JL coordinated data collection, prepared some of the descriptive analyses and commented on drafts of the paper. SJ assisted with the literature review, data collection and initial descriptive analyses. NR contributed to data analysis, interpretation and writing of the paper. CF advised on statistical analyses and study design. EC assisted with data collection and interpretation of findings. CH and GT supervised the evaluation and contributed to the design and writing of the paper. All authors read and approved the final manuscript.

## Pre-publication history

The pre-publication history for this paper can be accessed here:

http://www.biomedcentral.com/1471-2458/12/489/prepub
